# Antiplatelet Therapy with Integrated Traditional Chinese and Western Medicine for Use in Myocardial Ischemia-Reperfusion Injury: A Review of Clinical Applications and Mechanisms

**DOI:** 10.1155/2021/7409094

**Published:** 2021-07-20

**Authors:** Yuxuan Li, Yan Li, Bin Li, Yang Liu, Jingqian Zhang, Wu Kuang, Jinjin Lu, Zheng Cao, Jie Cui, Zongjing Fan, Bin Guo, Dong Li

**Affiliations:** ^1^Beijing University of Chinese Medicine, Beijing 100078, China; ^2^Department of Cardiology, Dong Fang Hospital, Fengtai District, Beijing University of Chinese Medicine, Beijing 100078, China

## Abstract

Myocardial ischemia-reperfusion injury (MIRI) is common in patients with acute coronary syndrome (ACS) after PCI treatment, which seriously affects the efficacy of revascularization and hinders the postoperative recovery of patients; therefore, the current study is focused on determining effective methods in the treatment of MIRI. Antiplatelet therapy is a routine treatment for ACS, and its benefits for treating MIRI have been previously verified. With the development of traditional Chinese medicine (TCM), many TCM preparations are widely used in the clinic. Many basic and clinical studies have shown that TCM can be used together with antiplatelet drugs, and the safety and efficacy when TCM is included in the treatment are better than when antiplatelet drugs are used alone. This paper summarizes the current research progress of traditional Chinese medicine and Western medicine in the treatment of MIRI to provide a theoretical basis for further research and clinical treatment.

## 1. Introduction

Acute coronary syndrome (ACS) is one of the leading causes of death worldwide [1]. ACS occurs when the blood flow through the coronary artery decreases or stops, resulting in myocardial tissue damage ranging from ischemia to infarction. The main mechanism is the growth and rupture of atherosclerotic plaques, and a thrombus is then formed within the coronary artery [2]. Due to different pathophysiological mechanisms between NSTE-ACS and STEMI, they are separated into two groups: NSTE-ACS and STEMI. NSTE-ACS guidelines recommend pharmacological treatment for ischemia (by coordinating the regulation of oxygen supply and consumption) [3]. STEMI's treatment guidelines recommend the immediate use of reperfusion therapy (initial percutaneous coronary intervention (PCI) or thrombolysis utilization) to recanalize the coronary artery [4]. All guidelines recommend antiplatelet agents to be used for long-term treatment after reperfusion. Revascularization is necessary for patients with myocardial infarction and can effectively reduce the cardiomyocyte injury caused by myocardial ischemia, but nearly one-half of AMI patients suffer from myocardial ischemia-reperfusion injury (MIRI) after reperfusion [5]. MIRI can cause a re-enlargement of the myocardial infarction area, which can manifest as a reperfusion arrhythmia, myocardial stunning, no reflow, microvascular dysfunction, and so on [6–8]. The reduction in the size of myocardial infarction in MIRI is an important goal in improving the effects of revascularization, so finding an effective treatment is the focus of MIRI research.

Platelets are discoid anucleate hematopoietic cells with an average diameter of 1.5–3.0 mm. They are produced by megakaryocytes in the bone marrow, circulate in blood, and are cleared by the reticuloendothelial system [9]. These cells can participate in MIRI in a variety of ways, and they can secrete a large number of positive and negative factors involved in the development of MIRI or could have a continuous activation in the vascular lesions leading to MIRI [10]. It is worth the attempt to use antiplatelet therapy for treating MIRI patients.

From the philosophy of TCM, there is a deficiency of qi in the pathogenesis of MIRI, and this can lead to the development of blood stasis, and blood stasis further aggravates qi deficiency; therefore, blood stasis is one of the important pathological processes in MIRI [11]. Many basic and clinical studies have achieved good results in the treatment of MIRI, such as when using *Salvia miltiorrhiza*, *Ligusticum chuanxiong*, and safflower. Currently, TCM and Western medicine have increased the understanding of antiplatelet therapy. This paper summarizes the current clinical research in both TCM and Western medicine for the treatment of MIRI and provides a theoretical basis for further clinical research in the future.

## 2. Relationship between Platelets and Clinical Manifestations of MIRI

Blocking platelet aggregation during PCI in patients with an acute myocardial infarction (AMI) is the standard treatment for inhibiting intravascular coagulation and reducing stent rethrombosis. Aggregates of platelets, leukocytes, and other substances produced after a coronary endothelial injury can occlude microcirculatory vessels, causing MIRI in ACS patients undergoing PCI. Platelets are also the first blood cells to respond to MIRI. Therefore, the effect of antiplatelet therapy on MIRI after PCI has been a research focus. Platelets have multiple pathophysiological effects in response to MIRI.

One of the effects is platelet microthrombosis [12]. Some researchers have found that platelets can be activated and they adhere and aggregate to the ischemic myocardial site in the early stage of myocardial ischemia-reperfusion after myocardial ischemia and reperfusion in animal models [13–15], and the number of activated platelets is positively correlated with the time of myocardial ischemia, also supporting that platelets can be directly activated by myocardial ischemia. The platelet glycoproteins GPVI and IIb/IIIa play key roles in platelet adhesion and aggregation, and several basic studies [16, 17] showed a reduction in the size of a MI by inhibiting GPVI in a murine left anterior descending artery ligation reperfusion model. These studies suggest that therapeutic strategies targeting the platelet glycoprotein GPVI may be a valuable approach for the treatment of MIRI.

In addition, platelet aggregation is an early marker of AMI and is also related to cardiac no-reflow in patients with STEMI after PCI [18, 19]. Ren et al. [19] believed that the increase in platelet levels before PCI was independently correlated with the no-reflow phenomenon after PCI, and the increased platelet level may indicate increased odds of the no-reflow phenomenon in STEMI patients receiving PCI. What is complicated is that the data [16] show that, in the mouse model of MI using a 30 min ligation followed by 24 h reperfusion, the size of MI is not affected by the inhibition of platelet adhesion or aggregation, but with the improvement of perfusion, it inhibited platelet activation and reduced the area of AMI, suggesting that platelet activation may affect the microvascular system. Although the effect of this treatment is not due to antithrombotic effects, it can still be confirmed that platelets affect the microvascular reperfusion [16].

Aggregation of platelets with leukocytes is also an important mechanism of MIRI, and it has been demonstrated in animal models and clinical trials that MIRI can induce an increase in platelet neutrophil complexes [19–21]. Studies have found that the levels of platelet neutrophil complexes are positively correlated with the severity of injury, neutrophils can promote platelet transendothelial motility, and phosphorylation of vasodilator-stimulated phosphoprotein (VASP) can reduce platelet-to-neutrophil aggregate production in peripheral blood and reduce platelet-to-neutrophil aggregate production in the ischemic myocardium, ultimately reducing the extent of injury in MIRI [22]. Animal studies [23] have found that ischemic preconditioning can induce VASP phosphorylation to inactivate GP IIb/IIIa integrin receptors on platelets, which leads to a decrease in platelet-neutrophil complex formation in injured organs and, in turn, decreased MIRI extent.

Platelets are activated and play an important role after MIRI stimulation, platelet activation causes an increase in the mean platelet volume and an increase in the secretion of microvesicles (MVSs), apoptotic bodies, and exosomes, and various mediators carried by these extracellular vesicles, such as proteins, mRNAs, and microRNAs, are released [24, 25]. Activated platelet-released factors may play a cardioprotective role during ischemia [26], but there is strong evidence that they can aggravate reperfusion injury by a mechanism independent of angioembolization [27, 28]. Further studies are needed. It is known that when ACS occurs, platelets are activated, and the mean platelet volume is larger than in stable coronary heart disease (CHD) patients [29]. Patients with AMI also have increased levels of released platelet granules (P-selectin and CD63) [30] and increased plasma levels of vWF and serotonin [8, 31], suggesting that platelets play an important role in myocardial injury.

Therefore, with the improved understanding of MIRI, it is recognized that platelets play important roles in the pathophysiology of MIRI and that antiplatelet therapy can inhibit the aggregation of platelets within the myocardium and inhibit the release of vasoconstrictive substances after platelet activation, indicating its great potential for MIRI therapy and that it is an important method for the treatment of MIRI in modern medicine.

## 3. Modern Antiplatelet Therapy for MIRI

Antiplatelet drugs are usually divided into anti-thromboxane A2, ADP receptor antagonists, PDE3 inhibitors, platelet GP IIb/IIIa receptor antagonists, and so on.

Dual antiplatelet therapy (DAPT) is the cornerstone of the treatment of ACS. Aspirin and clopidogrel are the most widely used antiplatelet drugs in the world. The CURE trial [32] evaluated the efficacy of aspirin (75 to 325 mg daily) combined with a loading dose of clopidogrel (300 mg loading followed by 75 mg daily) and aspirin alone for 9 months in patients with NSTE-ACS. The trial found that DAPT significantly reduced overall mortality within 24 h of symptom onset in patients with NSTE-ACS but was associated with an increased risk of bleeding compared with patients receiving clopidogrel alone. The COMMIT trial [33] also demonstrated that the addition of clopidogrel (75 mg per day) to aspirin (162 mg per day) therapy for 4 weeks after hospitalization safely reduced mortality and the risk of major cardiovascular events in patients with AMI. Khan et al.'s study [34] also confirmed that DAPT is indeed effective in preventing myocardial injury, preventing the expansion of myocardial infarction size, and reducing the risk of adverse cardiovascular events in patients after PCI, but there is a certain risk of bleeding. Antiplatelet therapy alone reduces bleeding events after PCI, whereas long-term DAPT reduces cardiovascular risk and prevents MIRI at the cost of increased bleeding events.

Guidelines [3, 4] recommend low-dose aspirin (75–100 mg) and a P2Y12 receptor antagonist after PCI in patients with AMI. Prasugrel 10 mg once daily is recommended for P2Y12 receptor antagonists and ticagrelor 90 mg twice daily and clopidogrel 75 mg once daily for 12 months when ticagrelor is not available to promote recovery from myocardial injury.

The choice of P2Y12 receptor antagonists was based on clinical trial selection. The PLATO trial [35] treated ACS patients for 33 months with aspirin (75–100 mg daily) in combination with either ticagrelor (180 mg loading dose, 90 mg twice daily) or clopidogrel (300 to 600 mg loading dose, 75 mg daily thereafter). The primary composite efficacy endpoint was achieved in 9.8% and 11.7% of patients in the final ticagrelor and clopidogrel arms, respectively, with no apparent difference in efficacy between the arms. Aspirin (75–162 mg) combined with prasugrel (60 mg loading dose and 10 mg maintenance daily) or clopidogrel (300 mg loading dose and 75 mg maintenance daily) in patients with ACS was continued for 6 to 15 months, with no significant difference in overall mortality between the two treatment groups [36].

MIRI is an important factor affecting the expansion of myocardial infarct size. DAPT may reduce myocardial infarct size by treating MIRI. Other antiplatelet agents can also be combined with DAPT to prevent against myocardial injury, such as GP IIb/IIIa inhibitors. Three GP IIb/IIa inhibitors are abciximab, tirofiban, and eptifibatide. Among them, abciximab is the most studied GP IIb/IIa inhibitor [37]. Basic research [27] found that GP IIb/IIIa inhibitors have the potential to treat MIRI. Researchers injected platelets from healthy volunteers and patients with AMI into rat hearts, and platelet perfusion in patients with AMI increased the coronary resistance and myocardial injury. The P2Y12 receptor antagonist ticagrelor and the GP IIb/IIa receptor blocker abciximab can prevent such injuries, suggesting that the early inhibition of platelet activation may be an effective method.

A meta-analysis evaluated the safety and efficacy of GP IIb/IIIa inhibitors during PCI. Forty-eight studies involved more than 33,000 patients. In short, the use of GP IIb/IIa inhibitors after PCI has been shown to be beneficial, as shown by a reduction in the 30-day mortality, nonfatal myocardial infarction, and emergency revascularization. However, this benefit is accompanied by a substantial risk of bleeding. The benefit disappeared 6 months after treatment [38]. Overall, the use of GPIIb/IIIa inhibitors as well as other antiplatelet agents was not randomized, and the addition of aspirin and P2Y12 receptor antagonists may increase the risk of bleeding. Due to the better efficacy and onset time of P2Y12 inhibitors, the use of other antiplatelet agents in ischemia-reperfusion is decreasing with the increased use of P2Y12 inhibitors.

## 4. Treatment of MIRI with Antiplatelet Therapy Using Integrative Medicine Therapy

Currently, 12 months of DAPT is commonly used in the clinic to prevent MIRI after PCI in patients with ACS. There are meta-analyses [39] which indicated that a treatment duration of 6 or 12 months had no significant difference on the impact of patients' risk of adverse cardiovascular events and that a longer period of DAPT increases the risk of bleeding, so how to adjust the time of DAPT and manage patients' risk factors is important. The above results also suggest that how to achieve adequate and effective antiplatelet therapy at 3–6 months of DAPT is important for patients' later benefit, while combining other antithrombotic agents, such as GP IIb/IIIa inhibitors, may increase the risk of bleeding. TCM formulations are able to reduce myocardial injury through multiple targets; therefore, the research into TCM formulations combined with Western antiplatelet agents such as DAPT on MIRI deserves further attention (Table 1).

### 4.1. Compound Danshen Dripping Pills

Compound Danshen Dripping Pill (CDDP) is a proprietary Chinese medicine based on TCM theory and modern medical technology. It regulates qi, relieves pain, promotes blood circulation, and removes blood stasis. Radix *Salviae miltiorrhizae*, *Panax notoginseng*, and borneol are the main components of CDDP, in which *Salvia miltiorrhiza* can activate blood circulation, remove blood stasis, and improve myocardial blood supply. *Panax notoginseng* can stop bleeding, invigorate blood circulation, remove blood stasis, and relieve pain. Borneol reduces swelling and relieves pain.

From the point of view of modern pharmacology, CDDP has the effects of antiplatelet aggregation and antithrombosis, reducing blood viscosity, protecting against reperfusion injury, and improving microcirculation. The main active components are salvianolic acid B and *Panax notoginseng* saponins [54]. A recent meta-analysis [40] included a number of clinical studies to explore the effect of CDDP on the hemorheology of CHD after PCI. CDDP significantly reduced MACE events after PCI (*P* < 0.00001). Compared with PCI alone, CDDP combined with PCI significantly reduced WBV, PV, and EAI (*P* < 0.00001), suggesting that CDDP can reduce the risk of postoperative PCI and treat MIRI by inhibiting platelet thrombosis and anticoagulation.

A meta-analysis [41] including 16 RCTs, after comparing the treatment groups using CDDP (10 pills, tid) combined with aspirin (50–150 mg daily) with the aspirin only group, concluded that CDDP combined with aspirin is effective in increasing the efficacy of CHD patients, reducing the rate of platelet aggregation, inhibiting platelet aggregation, and reducing thromboxane formation. The clinical study of Yan [42] included 180 patients with no reflow after PCI. The experimental group was treated with CDDP with antithrombosis and antiplatelet aggregation therapy. The TIMI blood flow grade of myocardial infarction was compared between the two groups. The experimental group had an improved blood circulation and a higher TIMI grade than the control group (*P* < 0.05). It is suggested that CDDP can significantly improve the myocardial microcirculation in patients with no reflow after PCI. DAPT combined with antithrombotic therapy is often adopted in the perioperative period after PCI of acute MI to prevent MIRI. The clinical trial designed by Wei et al. [43] included AMI patients on the basis of DAPT combined with CDDP in the perioperative treatment group, the control group was DAPT combined with bivalirudin, and the combination group was DAPT combined with bivalirudin and CDDP. The results of the study showed that the combination was able to improve cardiac function, optimize hemorheology, inhibit inflammation, and improve endothelial function in AMI patients after PCI.

Clinical studies [44] have found that CDDP can be combined with DAPT to treat patients with unstable angina, and after 3 weeks of treatment, it was found that CDDP combined with DAPT was most effective and was able to regulate the platelet aggregation rate and thromboxane B2. In the above clinical studies, CDDP was combined with antiplatelet drugs. The results showed that the combination of the two can promote and increase clinical efficacy, and the mechanisms need further study.

### 4.2. Naoxintong Capsules

Naoxintong capsules are based on the theory of TCM, which are made from the Buyang Huanwu decoction. It has the effect of replenishing qi and activating blood circulation, removing blood stasis, and dredging collaterals. Buyang Huanwu decoction is a clinically recognized prescription for the treatment of CHD with qi deficiency, blood stasis syndrome, and cerebrovascular disease [55].

Naoxintong capsules can reduce myocardial injury in patients with CHD. It has been found that taking Naoxintong capsules one week before PCI can reduce the levels of serum hs-CRP, CK-MB, and cTnI after PCI, reduce the myocardial injury caused by PCI, and reduce the damage caused by MIRI [45]. There may be many mechanisms for Naoxintong in the treatment of MIRI. Studies [56] have shown that Naoxintong capsules can inhibit proinflammatory macrophages (M1 macrophages) and neutrophil infiltration by inhibiting the activation of the inflammatory cytokines IL-1*β* and NLRP3 and improve cardiac function in mice with MIRI. There are also basic studies [57] that Naoxintong capsules can inhibit the platelet aggregation rate, prolong the clotting time, improve the hypercoagulable state of blood, and protect against ischemia in myocardial tissue in rats with myocardial ischemia, which can provide a theoretical basis for Naoxintong capsules in the clinical prevention and treatment of MIRI.

Huan [58] discussed the therapeutic effect of Naoxintong combined with DAPT in the prevention and treatment of coronary microembolism caused by autologous microthrombosis in rats. The results showed that Naoxintong capsules regulated the balance between hemorrhage and coagulation through multiple pathways and targets. They can significantly reduce CME% of coronary microembolism in model rats and reduce the bleeding risk in duplex antiplatelet therapy. The mechanism is that Naoxintong capsules can effectively regulate the levels of proinflammatory and anti-inflammatory factors, serum endothelin-1 (ET-1), and endothelial nitric oxide synthase (e-NOS), improve vascular endothelial function, and inhibit platelet aggregation.

Clinical studies have found that the combination of Naoxintong capsules with aspirin can provide optimized antiplatelet effects in patients with cardiovascular and cerebrovascular diseases. The study [46] included 145 patients with cardiovascular and cerebrovascular diseases (diabetes, hypertension, CHD, and cerebral infarction). The curative effect of the experimental group combined with aspirin was better than that of aspirin alone, and there were no increases in adverse reactions. CYP2C19∗2 is the most common genetic variation and is related to the bioavailability of clopidogrel active metabolites, antiplatelet effects, and variability of clinical results [59]. It can lead to a higher incidence of cardiovascular events in patients with a low CYP2C19 function after PCI [60], while Naoxintong [47] can be combined with clopidogrel to treat ACS patients, which has a significant effect on platelet aggregation, does not increase the risk of bleeding, and provides long-term clinical benefits for patients with CYP2C19∗2 gene mutations [61].

There are many mechanisms of Naoxintong in the treatment of MIRI, such as anti-inflammatory effects, antiplatelet aggregation, and improvement of vascular endothelial function that may be involved in the treatment of MIRI [62], but previous studies have shown that platelets play an important role in MIRI. Naoxintong combined with antiplatelet drugs can produce an improved effect, and the efficacy and safety of its combined use are worthy of further clinical research.

### 4.3. Danhong Injection

Danhong injection (DHI) is composed of *Salvia miltiorrhiza* and safflower, which has the effect of promoting blood circulation, removing blood stasis, and dredging meridians and collaterals. It mainly contains acids, tanshinones, flavonoids, and other chemical components [63]. By comparison with the control substance [64], it was determined that the material basis of DHI in the treatment of MIRI rats was danshensu, protocatechuic aldehyde, tanshinol acids, etc.

In the theory of TCM, the pathogenesis of MIRI is a blood stasis block. DHI has the effect of promoting blood circulation and removing blood stasis, while the aforementioned methods have been shown to be effective treatments for MIRI. Basic studies have found that DHI can increase the maximum platelet aggregation time of rabbits, effectively reduce the rate of platelet aggregation, and inhibit thrombosis in rabbits [65]. DHI can also use a variety of G protein-coupled receptor pathways to significantly inhibit platelet activation, dissolve arterial thrombi, and improve dry gangrene [66]. After treatment with DHI, the indexes of whole blood viscosity, nailfold microcirculation, and platelet activation in patients with myocardial infarction were significantly better than those in the control group, suggesting that DHI can effectively improve the disturbance of microcirculation [48]. Basic studies suggest that DHI has a certain effect on MIRI. Chinese experts agree that DHI can protect against myocardial ischemia-reperfusion injury in many ways [67].

DHI is often used in combination with antiplatelet drugs in the clinic. In a clinical study [49], DHI combined with antiplatelet drugs was used to treat 70 patients with STEMI after PCI. Cardiac structure and function and the size of the myocardial infarction were quantitatively evaluated by cardiac nuclear magnetic resonance (NMR). DHI improved the perioperative myocardial infarction area and cardiac function in PCI patients presented on emergency. In the clinical trial designed by Chen-guang et al. [50], DHI combined with clopidogrel was used to treat ACS. CD62P and the GP IIb/IIIa receptor complexes decreased significantly in the combined treatment group (*P* < 0.05), and inflammatory factors and vascular endothelial function were improved to some extent. DHI can also interact with aspirin, and the combination of drugs can inhibit the activity of aspirin esterase, enhance the antiplatelet effect of aspirin, increase the serum concentration of salicylic acid, and enhance the anti-inflammatory activity of salicylic acid [68]. Network pharmacology studies have also revealed that DHI can improve aspirin resistance in many ways, such as through coagulation processes, inflammatory reactions, and metabolic regulation. DHI and antiplatelet drugs can promote the antiplatelet effect, and there are no data to prove that DHI increases the risk of hemorrhage. The benefits and risks of the interactions and mechanisms of DHI need to be further explored.

### 4.4. Sodium Tanshinone IIA Sulfonate Injection

Sodium tanshinone IIA sulfonate injection (STS) is prepared by the sulfonation of fat-soluble tanshinone IIA, which is the main active component of *Salvia miltiorrhiza*. It dilates the coronary arteries, promotes the scavenging of oxygen free radicals, improves microcirculation, and protects the myocardium. Clinically, it is often used in combination with antiplatelet drugs such as aspirin and clopidogrel in the treatment of cardiovascular diseases.

Studies in vitro and in vivo have confirmed that the pharmacological effects of STS include antilipid peroxidation to prevent MIRI from preventing platelet aggregation and improving hemorheology [69]. Maione [70] confirmed that STS can inhibit platelet aggregation induced by adenosine diphosphate in a dose-dependent manner. In the rat model of MIRI [71], compared with saline, STS could significantly decrease the TNI of model mice, and the heart rhythm of mice in the STS group was more stable, indicating that STS has a potential therapeutic effect on MIRI. Xiaoli and Jianjun [51] found that STS could inhibit platelet activation in patients with unstable angina. The results showed that the expression levels of the platelet activation markers CD63 and PAC-1 in patients treated with STS were significantly lower than those in the control group, indicating that STS can significantly inhibit platelet activation and function and improve clinical symptoms.

STS is widely used in clinics. In clinical studies [52], STS combined with DAPT was used to treat ACS patients. It was found that STS can significantly reduce blood viscosity and inhibit the level of oxidative stress, and the clinical effect was better. Tao studied the effect of STS on ACS patients undergoing PCI. It was found that the plasma viscosity, whole blood high shear viscosity, and platelet adhesion rate in the combined treatment group were lower than those in the control group (*P* < 0.05), and the postoperative MIRI was improved to some extent [53].

The study [72] included 484 patients with NSTE-ACS who were treated with STS combined with DAPT. It was found that the combination therapy had better efficacy and safety. When STS was used in combination with clopidogrel, it was found that the former could bind to platelet ADP receptors, inhibit the activity of the ADP receptor, effectively reduce platelet aggregation, and enhance the antiplatelet effect of clopidogrel [73].

## 5. Summary

MIRI is an important sequela affecting the curative effect after revascularization in patients with ACS. Platelet aggregation or platelet activation is involved in the development of MIRI. Antiplatelet therapy with Western medicine, as the standard treatment after revascularization, can inhibit platelet aggregation and platelet activation. Many studies have confirmed its benefit in MIR therapy. At present, aspirin combined with P2Y12 receptor antagonists is the main antiplatelet therapy. With the improvement in research, there are more P2Y12 receptor antagonists to choose from, and there are new antiplatelet drugs to supplement DAPT, but the clinical benefits for MIRI are usually accompanied by an increase in the risk of bleeding. Some studies also believe that there is no significant improvement in clinical symptoms. TCM preparations, guided by theories such as invigorating blood circulation and eliminating stasis in TCM, are usually combined with Western antiplatelet therapy in the clinic, which is more effective clinically than antiplatelet agents alone, probably through the interaction with antiplatelet agents, and some studies considered that it does not increase the risk of bleeding. The combination of TCM and Western medicine in the treatment of MIRI is promising with regard to optimizing the antiplatelet treatment regimen. Millions of patients currently accept the treatment of integrative medicine in China, and the effectiveness and safety of these treatments have been fully verified in the clinic. Further clinical and basic research is needed to clarify these mechanisms and the effectiveness and safety of the drugs.

## Figures and Tables

**Table 1:** Summary of randomized studies on the effects of TCM formulations on CHD.

Studies	Interventions	Dose	Duration (months)	Result	Mechanism
Li et al. [40]	*E*: CDDP + PCI + conventional treatment *C*: PCI + conventional treatment	CDDP: 10 pills, tid	3 months	Combined CDDP may reduce the incidence of MACE in patients	—
Wang [41]	*E*: CDDP + aspirin *C*: aspirin	CDDP: 10 pills, tid Aspirin: 50–150 mg daily	3 months	CDDP combined with aspirin is effective in increasing the efficacy of CHD patients	Reducing the rate of platelet aggregation, inhibiting platelet aggregation, and reducing thromboxane formation
Yan [42]	*E*: CDDP + PCI + aspirin *C*: aspirin	CDDP: 10 pills, tid Aspirin: 100 mg daily	3 months	CDDP improves cardiac function and reduces the incidence of major long-term cardiovascular events in patients	Improved myocardial microcirculation
Wei et al. [43]	*E*: DAPT + CDDP *C*1: DAPT + bivalirudin *C*2: DAPT + bivalirudin + CDDP	CDDP: 10 pills, tid	1 week	The combination group was able to improve cardiac function	—
Tsai et al. [44]	*E*: DAPT + CDDP *C*1: clopidogrel *C*2: DAPT	CDDP: 10 pills, tid Clopidogrel: 75 mg daily Aspirin: 100 mg daily	3 weeks	Better improvement of symptoms in patients with unstable angina in the combination group	By downregulating platelet aggregation rate and thromboxane B2
Bo and Hui [45]	*E*: DAPT + Naoxintong capsules *C*: DAPT	Naoxintong capsules: 1.6 g, tid	1 week	Attenuates myocardial injury after PCI	Through anti-inflammatory effects
Chen et al. [46]	*E*: Naoxintong capsules + aspirin *C*: aspirin	Aspirin: 100 mg daily Naoxintong capsules: 3 pills, tid	1 month	Inhibition of platelet activity by Naoxintong capsules is superior to aspirin alone	—
Ming et al. [47]	*E*: Naoxintong capsules + DAPT *C*: DAPT	Clopidogrel: 75 mg daily Aspirin: 100 mg daily Naoxintong capsules: 3 pills, tid	5 days	The combination achieves more desirable antiplatelet aggregation without increasing the risk of bleeding	—
Lengqin and Yinghong [48]	*E*: DHI + conventional therapy *C*: conventional therapy	DHI: 30 ml daily	14 days	DHI can effectively improve the microcirculation state	—
Qi [49]	*E*: DHI + conventional therapy *C*: conventional therapy	DHI: 40 ml daily	6 days	DHI reduces myocardial infarct size and has the effect of improving cardiac function in perioperative patients undergoing PCI	—
Chen-guang et al. [50]	*E*: DHI + conventional therapy *C*: conventional therapy	DHI: 40 ml daily	2 weeks	The effect of the experimental group was better	Improved levels of inflammatory factors in patients and the inhibition of platelet activation as well as improved endothelial function
Xiaoli and Jianjun [51]	*E*: STS + aspirin *C*: aspirin	STS: 16 ml daily Aspirin: 100 mg daily	2 weeks	STS can improve clinical symptoms	STS can significantly inhibit platelet activation function
Qing [52]	*E*: STS + DAPT *C*: DAPT	Aspirin: 100 mg daily Clopidogrel: 75 mg daily STS: 80 mg daily	4 weeks	STS can reduce angina recurrence	STS can improve the inflammatory response, reduce blood viscosity, and protect cardiomyocytes
Tao [53]	*E*: STS + DAPT *C*: DAPT	Aspirin: 100 mg daily Clopidogrel: 75 mg daily STS: 100 ml daily	40 days	STS is effective in improving outcomes in ACS patients undergoing PCI	—

*E*: experimental group; *C*: control group; tid: three times per day; CDDP: Compound Danshen Dripping Pill; PCI: percutaneous coronary intervention; DAPT: dual antiplatelet therapy; DHI: Danhong injection; STS: sodium tanshinone IIA sulfonate injection.
